# The Gut Microbiome and Its Impact on Mood and Decision-Making: A Mechanistic and Therapeutic Review

**DOI:** 10.3390/nu17213350

**Published:** 2025-10-24

**Authors:** Pierluigi Diotaiuti, Francesco Misiti, Giulio Marotta, Lavinia Falese, Giovanna Elisa Calabrò, Stefania Mancone

**Affiliations:** Department of Human Sciences, Society and Health, University of Cassino and Southern Lazio, 03043 Cassino, Italy; francesco.misiti@unicas.it (F.M.); giulio.marotta@unicas.it (G.M.); l.falese@unicas.it (L.F.); giovannaelisa.calabro@unicas.it (G.E.C.); s.mancone@unicas.it (S.M.)

**Keywords:** gut–brain axis, gut microbiome, affective disorders, cognitive impairment, dysbiosis, neuroinflammation, psychobiotics

## Abstract

**Background/Objectives**: The gut microbiome is increasingly recognized as a key modulator of central nervous system function through the gut–brain axis. Dysbiosis has been associated with neuropsychiatric disorders such as depression, anxiety, impulsivity, cognitive decline, and addiction. This review aims to synthesize mechanistic insights and therapeutic perspectives on how gut microbiota influence mood regulation, decision-making, and cognitive processes. **Methods**: A comprehensive narrative review was conducted using peer-reviewed articles retrieved from PubMed, Scopus, and Web of Science up to August 2025. Studies were included if they explored microbiota-related effects on behavior, mood, cognition, or decision-making using human or animal models. Emphasis was placed on molecular mechanisms, microbiome-targeted therapies, and multi-omics approaches. **Results**: Evidence indicates that gut microbiota modulate neurochemical pathways involving serotonin, dopamine, GABA, and glutamate, as well as immune and endocrine axes. Microbial imbalance contributes to low-grade systemic inflammation, impaired neuroplasticity, and altered stress responses, all of which are linked to mood and cognitive disturbances. Specific microbial taxa, dietary patterns, and interventions such as probiotics, prebiotics, psychobiotics, and fecal microbiota transplantation (FMT) have shown promise in modulating these outcomes. The review highlights methodological advances including germ-free models, metagenomic profiling, and neuroimaging studies that clarify causal pathways. **Conclusions**: Gut microbiota play a foundational role in shaping emotional and cognitive functions through complex neuroimmune and neuroendocrine mechanisms. Microbiome-based interventions represent a promising frontier in neuropsychiatric care, although further translational research is needed to define optimal therapeutic strategies and address individual variability.

## 1. Introduction

The human microbiome, comprising bacteria, viruses, fungi, and archaea, forms a mutually beneficial ecosystem with the host, supporting essential physiological functions such as metabolism, immune regulation, intestinal barrier integrity, and neurophysiological signaling through dense trophic and molecular communication networks [1,2,3,4,5,6,7,8,9,10,11,12,13,14,15,16]. Throughout this review, we explicitly name representative probiotic strains, e.g., *Lactobacillus rhamnosus* GG, *Lactobacillus plantarum* PS128, *Bifidobacterium longum* 1714, and *Saccharomyces boulardii*, alongside key prebiotic substrates such as inulin-type fructans, galacto-oligosaccharides (GOS), resistant starch, arabinoxylans, and β-glucans, referencing exemplar clinical and mechanistic studies where relevant. Key commensals with well-described beneficial effects include *Faecalibacterium prausnitzii* (a major butyrate producer with anti-inflammatory activity), *Akkermansia muciniphila* (supports mucus-layer integrity and metabolic homeostasis), *Lactobacillus rhamnosus* GG and *Lactobacillus plantarum* PS128 (psychobiotic strains linked to improvements in stress, mood, and performance), and *Clostridium butyricum* (butyrate-mediated neuroprotection); in addition, *Lactobacillus reuteri* has emerged as a promising target for mechanistic clinical trials in the gut–brain axis [12,17,18,19,20].

In particular, the gut microbial community contributes to immune cell homeostasis, enteric nervous system regulation, angiogenesis, vitamin synthesis, energy balance, xenobiotic metabolism, and pathogen exclusion [1,2,4,6,9,10,11,12,15]. When this complex ecosystem becomes imbalanced, commonly referred to as dysbiosis, it has been linked to conditions such as non-alcoholic fatty liver disease and inflammatory bowel disorders [21,22,23].

Through the gut–brain axis, microbial states correlate closely with central nervous system (CNS) signaling pathways that influence mood, cognition, and behavior [12,13,14,15,24,25,26]. Gut microbes actively modulate host neurotransmitter systems, including serotonin and dopamine, which are critically involved in emotional regulation and psychological well-being. It also contributes to the detoxification of xenobiotics, modulation of bile acids, and synthesis of neuroactive metabolites that influence both local and systemic physiology [27,28,29,30,31,32].

Imbalances in gut microbiota composition have been associated with irritability and mood instability, often mediated by cytokine signaling that reduces tryptophan availability and serotonin synthesis [33,34,35]. Diet-induced microbiota changes also affect lipid and energy metabolism, with downstream effects on emotional states [36,37,38,39,40]. Bidirectional gut–brain communication influences brain development, behavior, and cognitive performance across the lifespan [24,25,26].

Increasing evidence also connects gut dysbiosis with cognitive deficits and impaired decision-making, through mechanisms involving neurotransmitter imbalances, neuroinflammation, and metabolic disruptions [41,42,43,44,45,46,47,48,49]. Both human and preclinical studies suggest that microbial diversity is positively linked to cognitive function in aging, with Mediterranean dietary patterns and targeted supplementation (e.g., prebiotics, probiotics) showing particular promise [44,45,46,49,50,51].

Alterations in the gut microbiome have been implicated in diabetes-related cognitive changes and forms of dementia, with emerging attention to the role of the gut mycobiome [52,53,54,55].

### Scope and Rationale

Against this backdrop, numerous studies have begun to examine the relationship between microbial composition and physiological domains such as glucose regulation, systemic inflammation, intestinal barrier function, adipose metabolism, bile-acid and hormonal signaling, and xenobiotic biotransformation. These systems intersect with central neural circuits involved in stress, reward, and emotional processing [10,11,12,13,14,15]. Despite a rapidly expanding literature, current reviews seldom provide a mechanism-anchored synthesis that links named probiotic strains and defined prebiotic substrates to CNS-relevant human outcomes (stress, mood, attention/cognition, performance) through clearly articulated pathways (SCFAs/butyrate, barrier integrity, bile-acid and tryptophan metabolism, vagal/HPA and neuroimmune signaling). Evidence is often summarized at the genus/species level, blurring strain specificity and dosing/duration considerations, while heterogeneity in outcomes, small samples, short interventions, and incomplete control of modifiers (dietary fiber, baseline microbiome, sleep/circadian factors, medications, physical activity) limit comparability and translation. Mapping from preclinical signals to human endpoints and integrating emerging domains such as decision-making and attentional bias remain underdeveloped, with little emphasis on precision (who responds, under what conditions). To address these gaps, this review offers a named-agent, mechanism-first synthesis, explicitly connecting key strains (e.g., LGG, PS128, *B. longum* 1714, *S. boulardii*) and substrates (inulin-type fructans, GOS, RS2/RS3, arabinoxylans, β-glucans) to CNS-relevant outcomes while appraising study quality and sources of heterogeneity. For comprehensive overviews and background perspectives on microbiome–brain interactions, readers may also consult recent reviews and position papers [56,57,58,59]. This review seeks to integrate mechanistic and therapeutic perspectives on the gut microbiome’s impact on mood and cognition. Particular emphasis is placed on methodological quality, translational challenges, and future directions to help bridge the gap between basic research and clinical applications.

## 2. Materials and Methods

**Figure 1:**
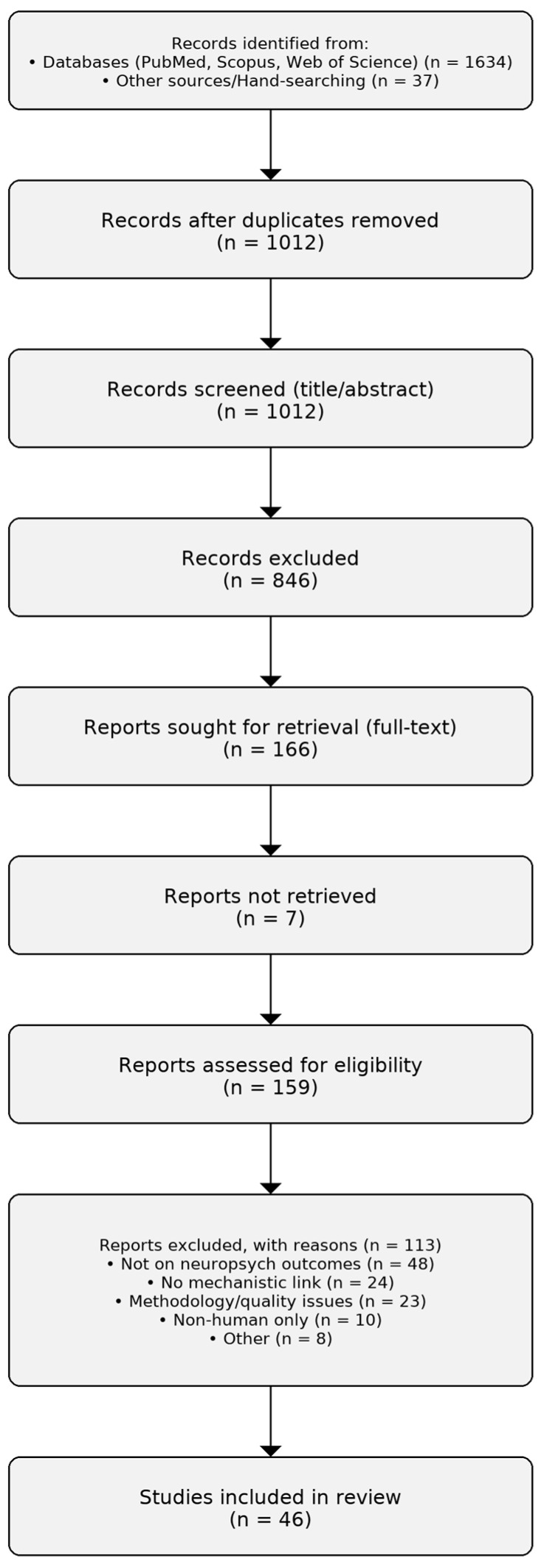
PRISMA-style flow diagram of study selection.

This narrative review was conducted by synthesizing evidence from peer-reviewed literature to explore the mechanisms through which the gut microbiome modulates mood, cognition, and decision-making. A comprehensive literature search was carried out across major academic databases including PubMed, Scopus, and Web of Science. The search included articles published from 1 January 2000 to 31 August 2025 and used keywords such as gut–brain axis, gut microbiota, mood disorders, cognitive function, neuroinflammation, psychobiotics, and fecal microbiota transplantation. In data extraction we systematically tracked named probiotic strains and prebiotic substrates (when reported at strain/substrate level) and recorded their primary putative mechanisms and outcome domains to harmonize terminology across sections. Both preclinical (animal model) and clinical (human subject) studies were considered, with particular emphasis on mechanistic investigations and therapeutic interventions. To enhance transparency, we summarize the study selection process in a PRISMA-style flow diagram (Figure 1), including numbers identified, screened, excluded with reasons, and included in the narrative synthesis. We followed a PRISMA 2020-style flow [60] to enhance transparency of the study selection process, while conducting a narrative (non-systematic) review without meta-analysis. Screening proceeded in two stages: first, titles and abstracts were screened to remove clearly ineligible records; second, full texts were retrieved and assessed for eligibility, with reasons for exclusion documented (e.g., not CNS-relevant outcomes, no mechanistic link, in vitro/cell-line only, methodological limitations, other). We excluded in vitro and cell-line-only studies from screening and data extraction because they do not capture whole-organism interactions required for CNS-relevant outcomes (e.g., neuroendocrine, immune, vagal/HPA pathways). Findings from in vitro work were considered only as narrative mechanistic background when directly supportive of in vivo pathways; they were not counted as included records and were not part of the PRISMA flow.

Inclusion criteria comprised (1) studies examining gut microbiota influence on neuropsychological or behavioral outcomes; (2) use of multi-omics technologies (e.g., metagenomics, metabolomics, metaproteomics); (3) intervention studies involving probiotics, prebiotics, psychobiotics, or dietary manipulation; (4) publications in English from peer-reviewed journals. Exclusion criteria included (1) non-peer-reviewed articles; (2) opinion pieces lacking empirical data; and (3) studies focused solely on gastrointestinal outcomes unrelated to neurobehavioral effects.

The selected literature was critically appraised for methodological rigor, sample size, reproducibility, and translational relevance. Special consideration was given to studies that incorporated causality models (e.g., germ-free animal designs, fecal transplantation) and those using integrated omics or neuroimaging to elucidate host–microbiome interactions. No formal meta-analysis was performed due to heterogeneity in methodologies, outcome measures, and microbial assessment techniques across studies.

## 3. Results

Gut microbes are known to synthesize a range of neuroactive compounds, such as gamma-aminobutyric acid (GABA), and to modulate immune–endocrine mediators that are highly relevant to mood disorders including depression and anxiety. These effects may be mediated by low-grade inflammation and reduced tryptophan availability, which limit central serotonin synthesis and alter emotional regulation [61,62,63,64,65,66,67,68,69,70]. Microbial imbalances, particularly dysbiosis, have been implicated in the disruption of sleep patterns and circadian rhythms, thereby amplifying symptoms related to chronic stress and emotional instability [64,65,66,69,70,71]. Taken together, this body of evidence supports the notion that the gut microbiome is a critical modulator of neurochemical activity and emotional regulation, playing a central role in the etiology of mood disorders [67,68,69,70].

An overview of key agents is provided in Box 1, with detailed mechanisms and outcomes summarized in Table 1 below.

Box 1 **Key probiotics and prebiotics referenced in this review.**

Probiotics (exemplar strains): *Lactobacillus rhamnosus* GG (ATCC 53103); *Lactobacillus plantarum* PS128; *Bifidobacterium longum* 1714; *Saccharomyces boulardii*.

Prebiotics (exemplar substrates): Inulin-type fructans; Galacto-oligosaccharides (GOS); Resistant starch (types 2–3); Arabinoxylans; β-glucans.

Principal mechanisms (overview): Short-chain fatty acid generation (notably butyrate), reinforcement of epithelial barrier and mucus layer, bile-acid and tryptophan pathway modulation, immune signaling, and vagal/neuroendocrine interfaces.

Importantly, the influence of the gut microbiota is not confined to the gastrointestinal tract. Instead, microbial communities exert systemic effects on distant organs such as the lungs, heart, and liver, as well as on the central nervous system, via endocrine, immune, and neural pathways [17,72,73,74,75]. A compelling illustration of this systemic interplay is offered by fecal microbiota transplantation (FMT), which has demonstrated causal influence on host phenotypes and is currently being explored for its potential impact on neurobehavioral conditions, including Alzheimer’s disease and inflammatory pain syndromes [76,77,78,79,80]. These associations also extend to the renal system, particularly within the framework of the gut–kidney axis, where microbial states have been linked to chronic kidney disease progression [81,82].

From a mechanistic perspective, microbial pathways play a vital role in the synthesis and regulation of neurochemicals such as GABA, serotonin, dopamine, and their respective precursors. These metabolites influence receptor expression and CNS signaling, particularly via vagal pathways and modulation of precursor bioavailability [83,84,85,86,87,88,89,90,91]. Notably, experimental studies using germ-free animal models have revealed profound shifts in fecal and serum neurotransmitter levels, underscoring the foundational role of microbiota in setting neurochemical baselines [87,88,89,90]. In addition, emerging research has begun to investigate the immunomodulatory effects of gut microbes in the context of psychiatric disorders such as schizophrenia, further broadening the relevance of microbiota to mental health [92].

A substantial body of literature has also established a link between low-grade systemic inflammation and mood disorders, including depression and bipolar disorder. These conditions frequently present with disruptions in glutamatergic signaling, and it is noteworthy that many psychotropic medications exhibit ancillary anti-inflammatory properties [93,94,95,96,97,98,99]. Such findings highlight plausible biological bridges connecting dysbiosis, immune dysfunction, and affective symptomatology.

The microbiota–gut–brain axis represents a highly integrated system that includes enteroendocrine signaling, neural pathways (notably involving the vagus nerve), and immune communication [100,101,102,103,104,105,106]. Dysbiosis within this axis has been proposed not only as a biomarker but also as a modifiable therapeutic target across a range of neurological and psychiatric disorders, including Alzheimer’s disease, Parkinson’s disease, irritable bowel syndrome (IBS)-related comorbidities, and bipolar disorder. Mechanistic pathways likely involve toll-like receptor 4 (TLR4) activation, lipopolysaccharide (LPS)-mediated signaling, intestinal barrier integrity, and neuroimmune crosstalk [101,102,103,104,105].

### 3.1. Effects on Decision-Making and Cognition

Beyond descriptive associations, converging evidence supports causal routes by which gut-derived signals may shape decision-making processes. First, short-chain fatty acids (SCFAs), notably butyrate, modulate microglial tone, synaptic plasticity, and blood–brain barrier integrity, with downstream effects on prefrontal efficiency and cognitive control; these pathways provide a biological bridge from fiber-fermenting prebiotics (e.g., inulin-type fructans, GOS, RS2/RS3) and butyrogenic commensals to performance on attention/executive tasks and reduced negative attentional bias in humans [51,52,53,54,55,61,62,63,64,65,78,79,80,81,82,83,84,85,86]. Second, tryptophan metabolism links the microbiome to central serotonergic and kynurenine pathways that influence valuation, impulsivity, and flexibility; in parallel, bile-acid signaling via FXR/TGR5 interfaces with dopaminergic and energy-balance networks relevant to frontostriatal decision systems [78,79,80,81,82,83,84,85,86,93,94,95,96,97,98,99]. Third, vagal afferents and HPA-axis dynamics transmit interoceptive and stress signals that bias risk/effort trade-offs and executive control, offering a plausible route for psychobiotic strains (e.g., *Lactobacillus rhamnosus* GG, *L. plantarum* PS128, *Bifidobacterium longum* 1714, *Saccharomyces boulardii*) to influence perceived stress, attentional control, and task engagement [12,17,18,19,20,100,101,102,103,104,105,106]. Immune–inflammatory mediators (e.g., low-grade cytokine activity) alter corticostriatal function and reward processing, providing a mechanistic rationale for observed links between microbiome modulation, inflammation lowering, and shifts in decision-relevant behaviors (e.g., vigilance, cognitive persistence) [78,79,80,81,82,83,84,85,86,93,94,95,96,97,98,99,100,101,102,103,104,105,106]. Taken together, these pathways outline a mechanism-anchored model in which named strains and defined prebiotic substrates (see Box 1 and Table 1) target tractable biological levers that align with task-level outcomes central to decision-making and cognition in humans [12,17,18,19,20,51,52,53,54,55,61,62,63,64,65].

### 3.2. Influence on Cognitive Function

Molecular routes link microbes to cognition. Converging evidence indicates that short-chain fatty acids (SCFAs), especially butyrate, promote synaptic plasticity, microglial homeostasis, and blood–brain barrier integrity, supporting attention/executive performance and memory; these effects mechanistically connect butyrogenic commensals (e.g., *Faecalibacterium prausnitzii*, *Clostridium butyricum*) and prebiotics that drive SCFA production (inulin-type fructans, GOS, RS2/RS3, arabinoxylans, β-glucans) to human cognitive endpoints [51,52,53,54,55,61,62,63,64,65,78,79,80,81,82,83,84,85,86].

In parallel, tryptophan–serotonin/kynurenine and bile-acid (FXR/TGR5) pathways interface with frontostriatal dopamine and cortical networks relevant to working memory, flexibility, and valuation, while vagal/HPA-axis signaling and immune–inflammatory tone (low-grade cytokines) further bias cognitive control and processing efficiency [78,79,80,81,82,83,84,85,86,93,94,95,96,97,98,99,100,101,102,103,104,105,106]. Several taxa/strains modulate neurotransmitters (e.g., GABA, glutamate) and stress biology in ways consistent with cognitive benefits: *L. rhamnosus* GG, *L. plantarum* PS128, *B. longum* 1714, and *S. boulardii*; *Akkermansia muciniphila* (barrier/mucus layer) and emerging *L. reuteri* add mechanistic plausibility via barrier and neuroimmune pathways [12,17,18,19,20,100,101,102,103,104,105,106]. These routes align with the named-agent synthesis in Box 1/Table 1 and are consistent with neuroimaging and multi-omics links between microbial features and brain networks underlying cognition [20,100,101,102,103,104,105,106,107,108,109,110,111]. Additional cohort- and review-level evidence complements these findings and frames cognitive outcomes within broader microbiome–behavior links [112].

The pioneering work of Foster & McVey Neufeld [107] highlights the gut–brain axis’s role in regulating anxiety and depression, setting the stage for a broader understanding of the microbiome’s impact on higher cognitive functions such as reasoning, attention, memory, decision-making, learning, and problem-solving. Mayer et al. [108] further cement the paradigm shift in neuroscience by emphasizing the gut–brain axis’s significant influence on cognitive function.

Recent neuroimaging findings by Liu et al. [20] update our understanding of the gut microbiota’s interaction with cognitive function, while Liang et al. [109] integrate multi-omics data to elucidate the gut microbiome’s effects on cognitive function and brain structure. The regulation of Alzheimer’s disease pathologies and cognitive disorders through gut microbiota-mediated neuroinflammation, as demonstrated by Bairamian et al. [110], marks a critical advance in our understanding of neurodegenerative diseases. Wasser et al. [111] explore the associations between gut microbiota and cognitive performance in Huntington’s disease, offering insights into clinical outcomes.

The connection between Western diet consumption, the microbiome, and cognitive impairment presented by Noble et al. [42] illustrates the nutritional and lifestyle factors contributing to gut–brain dysbiosis. Smith et al. [113] introduce an intriguing association between gut microbiome diversity and sleep physiology, suggesting a broader physiological relevance of the microbiome. The emerging field of neuromicrobiology, as proposed by Miri et al. [114], and the insights into the microbiome’s effect on synaptic plasticity expand the scope of microbiome research into neurometabolic processes and synaptic malleability [115].

Investigations into specific interventions, such as the impact of American Ginseng on mood and cognition [116] and the psychological benefits of LGG probiotic supplementation [18], offer promising evidence of modulating the gut microbiome to enhance cognitive and psychological health. Also, the relationship between wine consumption, diet, and microbiome modulation in Alzheimer’s disease [51] opens a novel avenue for dietary interventions in neurodegenerative disease management.

This body of research provides compelling evidence that the gut microbiome significantly influences brain function and structure, cognitive performance, and neurological health outcomes. It underscores the potential of microbiome-targeted interventions as therapeutic strategies for cognitive disorders, psychological status, and overall brain health, marking a significant shift in our approach to neuroscience and mental health.

### 3.3. Impact on Risk Assessment

In this review, risk assessment denotes decision-making under risk, i.e., choices among options with explicit, known probabilities and outcome magnitudes, and, where noted, decision-making under ambiguity, where probabilities are only partially specified. Empirical work operationalizes these constructs through lottery/expected-value tasks, probability-discounting paradigms, and the Balloon Analogue Risk Task (BART) for risk-taking; the Iowa Gambling Task (IGT) primarily indexes decision-making under ambiguity rather than pure risk. Within this framework, signals attributed to psychobiotic strains (e.g., *Lactobacillus rhamnosus* GG, *L. plantarum* PS128, *Bifidobacterium longum* 1714, *Saccharomyces boulardii*) and prebiotics (inulin-type fructans, GOS, RS2/RS3, arabinoxylans, β-glucans) are interpreted via mechanism-anchored pathways, notably SCFA/butyrate effects on microglia and synaptic plasticity, barrier integrity, tryptophan–serotonin and bile-acid (FXR/TGR5) signaling with downstream dopaminergic influences, and vagal/HPA–immune interfaces that modulate arousal and stress responsivity, each of which can shift risk preference, probability weighting, and choice consistency on these tasks [12,17,18,19,20,51,52,53,54,55,61,62,63,64,65,78,79,80,81,82,83,84,85,86,93,94,95,96,97,98,99,100,101,102,103,104,105,106]. Accordingly, where we report changes in “risk taking” we refer specifically to task-level indices under known or partially known probabilities rather than generic behavioral descriptors; results are cross-referenced to Box 1/Table 1 to maintain traceability to the underlying named agents and mechanisms [12,17,18,19,20,51,52,53,54,55,61,62,63,64,65].

Fecal microbiome analysis has identified decreased abundance of Firmicutes, specifically Faecalibacterium, in patients presenting with bipolar disorder, which also correlated with self-reporting symptom severity, indicating a potential link between gut microbiome composition and psychiatric symptoms [50]. Studies have demonstrated that the gut microbiome influences dopamine levels in the frontal cortex and striatum in rodents, brain areas involved in executive functions, suggesting a potential mechanism through which the microbiome may impact risk-related behaviors [117]. Other findings suggest that targeting the microbiome may represent a novel approach for treating neurodevelopmental disorders such as autism, further emphasizing the potential role of the gut microbiome in shaping behaviors and psychiatric conditions [118]. Animal studies have also shown that the presence of the gut flora in rodents may impose behavioral changes with wider implications for psychiatric disorders in humans [119]. The gut microbiome has been implicated in various psychiatric conditions, including major depressive disorder, bipolar affective disorder, autism spectrum disorder, anxiety disorders, schizophrenia, and psychotic disorders, highlighting the broad impact of the microbiome on mental health [63].

Microbiome has been associated with metabolic syndrome, obesity, diabetes, dyslipidemia, and hypertension, suggesting a potential link between the microbiome and physiological factors that may contribute to psychiatric disorders [120,121]. Taken together, the pattern suggests modest, mechanism-consistent shifts in risk-sensitive choice, most plausibly mediated by stress/HPA attenuation, inflammation lowering, and serotonergic/dopaminergic pathway engagement, rather than a uniform change in “riskiness” per se, reinforcing the need for standardized, probability-explicit tasks in future trials [78,79,80,81,82,83,84,85,86,93,94,95,96,97,98,99,100,101,102,103,104,105,106].

### 3.4. Connection to Impulsive Behavior

In this review, impulsivity refers to a multidimensional tendency toward premature or poorly controlled action and can be conceptualized as both a trait (stable, dispositional) and a state (stress- or context-sensitive). We consider three principal facets that are commonly quantified in human and preclinical work: (1) motor impulsivity (deficits in response inhibition, typically assessed with go/no-go or stop-signal tasks); (2) choice impulsivity (a preference for smaller-sooner over larger-later rewards, measured via delay-discounting paradigms); and (3) reflection impulsivity (reduced information gathering before choice). This construct is distinct from risk-taking, which concerns choices under known or partially known probabilities (treated separately in our “Risk Assessment” section). Within this framework, gut-derived pathways—SCFA/butyrate effects on plasticity and microglial tone, tryptophan–serotonin/kynurenine and bile-acid signaling (FXR/TGR5) with downstream dopaminergic influences, and vagal/HPA–immune interfaces affecting arousal and stress responsivity—offer biologically plausible routes by which named probiotics (e.g., LGG, PS128, *B. longum* 1714, *S. boulardii*) and defined prebiotics (inulin-type fructans, GOS, RS2/RS3, arabinoxylans, β-glucans) could modulate task-level indices of impulsivity [12,17,18,19,20,51,52,53,54,55,61,62,63,64,65,78,79,80,81,82,83,84,85,86,93,94,95,96,97,98,99,100,101,102,103,104,105,106,122,123,124,125,126,127,128,129,130,131,132,133,134]. Broader syntheses and related primary studies further contextualize impulsivity-related outcomes within microbiome research [135,136,137,138,139,140,141].

The relationship between the gut microbiome and impulsive behavior is an area of growing interest in the field of neuroscience and psychology. Several studies have highlighted the potential impact of the gut microbiome on cognitive functions, decision-making, and impulsive behaviors [122,123,124,125,126,127,128,129]. The gut microbiome has been shown to influence various aspects of behavior, including impulsivity, attention, reward-learning, and locomotor response to novelty [130]. Alterations in the gut flora have been correlated with increased impulsivity and vulnerability to substance use disorders [131]. These findings suggest a potential link between the gut microbiome and impulsive behavior. Research has also indicated that the gut microbiome may covary with the processing in frontoparietal circuits associated with cognitive control and self-regulation, which are essential for regulating impulsive behaviors [132]. Studies in animal models have suggested that changes in the microbiome can be associated with alterations in cognition and behavior, including impulse transmission along the vagus nerve, which connects the intestinal tract and the central nervous system [133].

The microbiome has been implicated in the modulation of mood and affect, which are closely related to impulsive behavior [129]. Traits associated with sensitivity to drug stimuli in animals have been linked to alterations in the microbiome, further emphasizing the potential role of the gut microbiome in impulsive behaviors [134].

The potential impact of the gut microbiome on impulsive behavior has significant implications for various fields, including psychiatry, neurology, and addiction medicine. Understanding the mechanisms through which the gut microbiome influences impulsive behavior could lead to novel therapeutic interventions targeting the microbiome to modulate impulsive tendencies and improve cognitive control.

### 3.5. Role in Addiction and Substance Abuse

Several reviews and primary studies converge on the microbiome’s contribution to reward processing and substance-related phenotypes, offering frameworks that complement the targeted reports summarized below [135,136,137,138,139,140,141,142,143,144,145,146,147]. Forouzan, McGrew, and Kosten [148] delve into the relationship between negative affect, psychostimulant use and withdrawal, and the microbiome, providing insights into how disruptions in gut flora may exacerbate psychological distress associated with addiction. García-Cabrerizo et al. [130] further elucidate the microbiota–gut–brain axis’s regulatory capacity on reward processes, suggesting that gut microbiota may influence the brain’s reward circuitry, thereby affecting addictive behaviors. Russell et al. [149] review the gut microbiome’s role in substance use disorder, proposing that alterations in gut flora could influence the predisposition to and outcomes of addiction treatments. Cryan et al. [50] provide a comprehensive overview of the microbiota–gut–brain axis, emphasizing its significance in brain diseases and, implicitly, in the modulation of behaviors related to addiction. Research by Browning et al. [150] on the potential role of the gut microbiome in substance use disorders highlights the connection between gut dysbiosis, illicit drug use, and the immune system’s role in addiction. Similarly, studies like that of Molavi et al. [151] investigate the effects of probiotic supplementation on opioid-related disorders, suggesting that modifying the gut microbiota could offer therapeutic benefits in addiction treatment. Emerging research, such as that by Fu et al. [152], points to the microbiome–gut–brain axis as a promising therapeutic target for substance-related disorders, indicating that interventions aimed at restoring gut microbial balance may mitigate addiction behaviors. Studies like that of Ren & Lotfipour [153] show that the gut microbiota composition can vary significantly with opioid use, impacting circulating levels of hormones such as leptin and oxytocin, which are known to influence addiction pathways. The role of diet in modulating the gut microbiota and, consequently, addiction behaviors cannot be overstated, as evidenced by Yang et al. [154], who explore the impact of dietary nutrients on gut flora. This line of research suggests that nutritional interventions could alter the gut microbiome in a manner that supports addiction recovery processes.

The literature underscores the gut microbiome’s profound influence on addiction behaviors through various pathways, including the modulation of the gut–brain axis, immune responses, and dietary impacts on gut flora. These findings pave the way for innovative approaches to addiction treatment, emphasizing the potential of microbiome-targeted therapies to complement traditional interventions. The complexity of the microbiome’ s role in addiction underscores the need for further research to unravel the intricate mechanisms by which the gut influences brain health and behavior, offering hope for more effective and personalized addiction treatment strategies.

### 3.6. Factors Shaping Microbiome Composition

The composition and functional diversity of the gut microbiome are highly dynamic and sensitive to a variety of internal and external influences. Among the most powerful of these are dietary patterns, which exert profound effects on microbial populations, modulating both inflammatory tone and overall disease risk. Diets rich in plant-based foods and dietary fibers, such as fruits, vegetables, legumes, and whole grains, have consistently been associated with favorable outcomes in terms of metabolic health and neurocognitive performance [37,40]. In contrast, diets low in fiber and high in refined sugars and saturated fats tend to promote dysbiosis and inflammation. Importantly, exposures during early life, including to allergens, nutritional components, and microbial environments, can shape the long-term trajectory of the gut microbiome, with lasting implications for health and behavior [155,156,157].

Beyond nutrition, psychological and pharmacological stressors represent additional key modulators of gut microbial ecology. Chronic stress, traumatic experiences, and the use of certain psychotropic or somatic medications can induce shifts in microbiota composition, leading to downstream effects on pain sensitivity, metabolic processes, and mental health across the lifespan [158,159,160,161,162,163,164,165]. Notably, early-life stress has been shown to exert long-term programming effects on both the microbiome and behavioral outcomes, reinforcing the concept of a sensitive developmental window for host–microbe interactions [161,162,163].

Pharmacological agents, particularly antibiotics but also non-antibiotic drugs, can significantly perturb microbial ecosystems too. Such disturbances often result in the depletion of beneficial taxa and reduced microbial stability. In experimental models, these ecological disruptions have even been causally linked to increased mortality, highlighting the critical role of microbial homeostasis for host resilience [166,167,168].

Another factor that influences microbiota composition is physical activity, especially when performed at high intensity. While moderate exercise can have beneficial effects, excessive exertion may transiently increase systemic inflammation and compromise intestinal barrier function, a phenomenon often referred to as “leaky gut”. However, this effect can be mitigated through targeted nutritional strategies and probiotic supplementation, which help buffer oxidative stress and promote adaptive recovery responses. Also, disruption of circadian rhythms, including irregular sleep patterns, has been shown to alter microbial diversity and composition, with downstream effects on hormonal regulation and emotional reactivity [19,169,170,171,172].

These findings underscore the multifactorial nature of microbiome regulation, suggesting that individualized interventions targeting diet, stress management, medication use, and physical activity could offer integrated strategies to promote gut and brain health.

### 3.7. Methods to Study Microbiome–Mood Links

The investigation of microbiome–brain interactions has benefited immensely from recent advances in multi-omics technologies, which allow researchers to unravel complex structure–function relationships within microbial ecosystems. Among the most widely used techniques are 16S rRNA gene sequencing, shotgun metagenomics, metatranscriptomics, metaproteomics, and metabolomics. These tools provide detailed insights into the taxonomic composition, gene expression patterns, protein synthesis, and metabolic profiles of gut microbiota [173,174,175,176,177,178,179,180,181,182].

To make sense of the massive and multidimensional datasets generated by these approaches, researchers increasingly rely on compositional data analysis techniques and machine learning models that can uncover hidden patterns and functional associations. In particular, network-based approaches and Gaussian process models are proving valuable in capturing dependencies across multiple omics layers, such as transcript–metabolite correlations or protein–taxon relationships [183,184]. However, for findings to be replicable and translatable, the standardization of analytical pipelines remains a critical priority. Certified workflows, quality control metrics, and harmonized data formats are essential for achieving cross-study comparability and advancing the field [185,186].

In addition to human cohort studies, animal models continue to serve as indispensable platforms for establishing causal relationships between microbial colonization patterns and behavioral phenotypes. Experimental manipulations, such as germ-free environments, microbial transfers, or selective antibiotic exposure, enable researchers to directly link microbiota configurations to changes in neurotransmitter levels, particularly serotonin and GABA, and corresponding alterations in affective and cognitive behavior [90,187,188].

While animal models provide critical insights into mechanistic pathways, translating these findings to human populations requires caution due to interspecies differences in microbial architecture, neurodevelopment, and behavior. On the human side, researchers often evaluate the impact of interventions such as antibiotics, prebiotics, probiotics, and controlled diets on emotional well-being and cognitive performance. These interventions are complemented by emerging strategies such as remote cognitive testing, which enhances the feasibility of studying neuropsychological outcomes even in vulnerable or hard-to-reach populations, including individuals with severe mental illness [189,190,191,192,193].

### 3.8. Therapeutic Approaches

Growing evidence supports the use of microbiome-targeted therapies as promising adjuncts in the treatment of mood and cognitive disorders. This includes not only mood and anxiety disorders but also emerging indications such as autism spectrum conditions, multiple sclerosis, and Parkinson’s disease, where preliminary evidence suggests that microbiome-based interventions may modulate disease trajectory. Among these, probiotics, defined as live microorganisms that confer health benefits to the host, have shown the capacity to modulate the gut–brain axis, particularly in relation to stress biology and emotional regulation. Clinical trials have reported improvements in mood and anxiety symptoms following probiotic or prebiotic supplementation, underscoring their potential role as safe and accessible interventions. For clarity, examples cited throughout include probiotics such as *Lactobacillus rhamnosus* GG, *L. plantarum* PS128, *Bifidobacterium longum* 1714, and *Saccharomyces boulardii*, and prebiotics such as inulin/GOS, resistant starch, arabinoxylans, and β-glucans (WHO; [194,195,196,197,198,199,200,201,202,203,204,205,206,207,208,209,210]). Also, fermented foods, which naturally contain probiotic strains, may serve as complementary strategies within broader dietary patterns.

Another therapeutic avenue is fecal microbiota transplantation (FMT), which involves the introduction of gut microbiota from a healthy donor to a recipient via endoscopic delivery or oral capsules. While FMT is already established as an effective treatment for recurrent Clostridioides difficile infection, it is now being actively investigated for its potential impact on neuropsychiatric and neurodegenerative conditions. Preliminary studies suggest promising outcomes, although further research is needed to clarify mechanistic pathways, long-term effects, and safety protocols [194,211,212,213].

In parallel, dietary composition, particularly the balance of macronutrients and availability of fermentable substrates, plays a critical role in shaping the gut microbiota and influencing host physiology. Adjusting diet can alter microbial metabolism, reduce inflammatory markers, and impact emotional states. Importantly, aligning dietary strategies with cultural and individual preferences improves adherence and enhances psychological outcomes, thereby maximizing therapeutic potential [195,196,197].

Emerging within this context is the concept of psychobiotics, a class of probiotics and prebiotics specifically selected for their impact on central nervous system function. Randomized controlled trials (RCTs) have documented psychobiotic-induced reductions in cortisol levels, perceived stress, and depressive symptoms, suggesting that these agents may serve as low-risk adjuncts or, in some cases, alternatives to conventional treatments [198,199,200,201,202,203,204,205]. However, for psychobiotics to gain broader clinical acceptance, challenges related to regulatory approval, quality assurance, and strain-specific efficacy must be systematically addressed.

## 4. Discussion

Across the evidence base reviewed, the most consistently studied probiotics include *Lactobacillus rhamnosus* GG, *L. plantarum* PS128, *Bifidobacterium longum* 1714, and *Saccharomyces boulardii*, while prebiotics most frequently linked to beneficial stress/mood and cognition-relevant outcomes include inulin-type fructans, GOS, resistant starch (types 2–3), arabinoxylans, and β-glucans [12,17,18,19,20,51,52,53,54,55,61,62,63,64,65]. Mechanistically, convergent pathways span short-chain fatty acid (SCFA) production, especially butyrate-barrier reinforcement, bile-acid and tryptophan signaling, immune tone modulation, and vagal/HPA-axis interfaces [78,79,80,81,82,83,84,85,86,93,94,95,96,97,98,99,100,101,102,103,104,105,106]. Taken together, these findings suggest strain- and substrate-specific effects rather than a generic “microbiome” signal, with small-to-moderate, population-dependent benefits most consistently observed for perceived stress, mood indices, and selected attentional/cognitive endpoints [51,52,53,54,55,61,62,63,64,65].

Most important signals from human studies: Trials using LGG and PS128 report reductions in perceived stress and inflammatory markers, with PS128 additionally showing benefits in performance/recovery contexts; *B. longum* 1714 demonstrates stress-attenuating and attentional effects in human experimental paradigms. For prebiotics, GOS shows the clearest human signal for cortisol modulation and reduced negative attentional bias in young adults, whereas inulin-type fructans and resistant starch (RS2/RS3) consistently act as bifidogenic/butyrogenic substrates that translate into improvements in stress proxies, inflammatory/metabolic markers, and GI function, all domains plausibly connected to affect and cognition [12,17,18,19,20,51,52,53,54,55,61,62,63,64,65,78,79,80,81,82,83,84,85,86]. These agent-specific profiles align with the synthesis presented in Box 1 and Table 1 in the Results.

Mechanistic coherence and translational plausibility: Across agents, the most plausible molecular levers for cognition are butyrate-centric SCFA signaling (epigenetic/BDNF-linked plasticity; BBB/microglia), tryptophan–serotonin/kynurenine and bile-acid crosstalk (with downstream effects on dopamine and cortical–striatal networks), vagal/HPA interfaces (stress responsivity and arousal), immune–inflammatory recalibration, and GABA/glutamate modulation. These pathways provide a coherent rationale for the cognitive signals seen with *L. rhamnosus* GG, *L. plantarum* PS128, *B. longum* 1714, and *S. boulardii*, and with prebiotics that increase butyrate (inulin-type fructans, GOS, RS2/RS3, arabinoxylans, β-glucans); commensals such as *F. prausnitzii* and Akkermansia contribute via butyrate production and barrier reinforcement [12,17,18,19,20,51,52,53,54,55,61,62,63,64,65,78,79,80,81,82,83,84,85,86,93,94,95,96,97,98,99,100,101,102,103,104,105,106]. Human neuroimaging and multi-omics studies add convergent validity by linking microbiome features to attention and decision-making networks, reinforcing a mechanism-anchored interpretation of cognitive findings in this review [20,100,101,102,103,104,105,106,107,108,109,110,111]. Evidence linking butyrate-centric metabolism and barrier integrity to improved affective outcomes is supported by studies showing shifts in neurotransmitter precursors and neuroimmune mediators (e.g., tryptophan/serotonin availability, cytokines) and by emerging work implicating vagal/HPA-axis signaling in stress responsivity [78,79,80,81,82,83,84,85,86,93,94,95,96,97,98,99,100,101,102,103,104,105,106]. Early neuroimaging and multi-omics studies in humans further connect microbial features with brain networks underlying attention, decision-making, and emotional regulation, supporting a mechanism-anchored view of psychobiotic and prebiotic actions [100,101,102,103,104,105,106].

Evidence integration across modalities: Converging evidence from human interventions, animal models, and multi-omics/neuroimaging strengthens the causal narrative linking gut signals to CNS-relevant outcomes. Named strains (LGG, PS128, *B. longum* 1714, *S. boulardii*) and butyrogenic prebiotics (inulin-type fructans, GOS, RS2/RS3, with supportive signals for arabinoxylans and β-glucans) align with butyrate-centric SCFA pathways, barrier support, tryptophan–serotonin/kynurenine and bile-acid crosstalk, and vagal/HPA interfaces that modulate stress responsivity, attention, and executive control [12,17,18,19,20,51,52,53,54,55,61,62,63,64,65,78,79,80,81,82,83,84,85,86,93,94,95,96,97,98,99,100,101,102,103,104,105,106]. Human neuroimaging and multi-omics links between microbial features and frontostriatal/prefrontal networks provide independent corroboration of these mechanisms in cognitive and decision-related domains [20,100,101,102,103,104,105,106,107,108,109,110,111]. These modalities triangulate small-to-moderate, mechanism-consistent effects on perceived stress, mood indices, attentional bias, and selected cognitive endpoints reported in the Results.

Named microbiome agents with plausible CNS benefits: Converging evidence indicates that selected probiotic strains (*Lactobacillus rhamnosus* GG-ATCC 53103, *L. plantarum* PS128, *Bifidobacterium longum* 1714, and *Saccharomyces boulardii*) are the most consistently studied in relation to stress/mood modulation and attentional/cognitive endpoints [12,17,18,19,20]. In parallel, the prebiotics most frequently linked to favorable CNS-relevant outcomes are inulin-type fructans, galacto-oligosaccharides (GOS), resistant starch (RS2/RS3), arabinoxylans, and β-glucans [51,52,53,54,55,61,62,63,64,65]. Mechanistically, these agents converge on SCFA production (notably butyrate), epithelial barrier reinforcement, bile-acid and tryptophan pathway modulation, and neuroimmune–neuroendocrine signaling (including vagal and HPA-axis interfaces), providing biologically grounded routes to central effects [78,79,80,81,82,83,84,85,86,93,94,95,96,97,98,99,100,101,102,103,104,105,106]. Beyond intervention agents, beneficial commensals with mechanistic plausibility for CNS impact include butyrate-producing *Faecalibacterium prausnitzii* and mucus-layer-supporting *Akkermansia muciniphila*, as well as butyrate-linked *Clostridium butyricum*; *Lactobacillus reuteri* is also emerging as a promising target for mechanism-first clinical trials within the gut–brain axis [12,17,18,19,20]. These taxa and substrates map onto reduced perceived stress and inflammatory tone, improvements in mood indices, and signals for attention/cognition.

Strengths of the included studies: Several features across the included literature increase confidence in the signals observed. First, a subset of human trials used named, strain-specified probiotics and well-defined prebiotic substrates, enabling mechanism-anchored interpretation and cross-study comparability (e.g., LGG, PS128, *B. longum* 1714; inulin-type fructans, GOS, resistant starch) [12,17,18,19,20,51,52,53,54,55,61,62,63,64,65]. Second, converging biological readouts, including inflammatory markers, endocrine indices (e.g., cortisol) and GI/barrier-related measures, map onto hypothesized pathways (SCFAs, barrier integrity, bile-acid and tryptophan signaling, vagal/HPA interfaces), reinforcing the mechanistic coherence of the clinical effects [78,79,80,81,82,83,84,85,86,93,94,95,96,97,98,99,100,101,102,103,104,105,106]. Third, an increasing number of studies incorporate multi-omics and/or neuroimaging approaches, which begin to link microbial features with brain networks and cognitive/affective endpoints, improving translational plausibility [100,101,102,103,104,105,106]. Evidence spans diverse populations and settings, which helps to assess robustness and boundary conditions for psychobiotic/prebiotic actions [12,17,18,19,20,51,52,53,54,55,61,62,63,64,65].

Where the signal is strongest vs. emerging: Among probiotics, LGG and PS128 show the most consistent reductions in perceived stress and inflammatory tone, with PS128 also supporting recovery/performance contexts; *B. longum* 1714 exhibits attentional and cortisol effects in human experimental paradigms. For prebiotics, GOS most clearly modulates cortisol and negative attentional bias in young adults, while inulin-type fructans and RS2/RS3 reliably drive bifidogenic/butyrogenic changes that couple to improvements in stress proxies, metabolic/inflammatory markers, and GI function, domains plausibly upstream of affect and cognition [12,17,18,19,20,51,52,53,54,55,61,62,63,64,65]. These patterns are coherent with the mechanistic levers summarized above and are echoed by systems-level studies relating microbial composition/function to brain networks and cognitive performance [20,100,101,102,103,104,105,106,107,108,109,110,111]. At the same time, heterogeneity in samples, duration, dosing/strain precision, and outcome measures explains residual variance and identifies priorities (standardized endpoints, biomarker anchoring, responder analyses) for the next generation of trials [93,94,95,96,97,98,99,100,101,102,103,104,105,106].

Clinical implications (near-term): Given current evidence, named strains (LGG, PS128, *B. longum* 1714) and prebiotics (GOS, inulin-type fructans, RS2/RS3; with supportive but more limited signals for arabinoxylans and β-glucans) are plausible adjuncts for individuals with stress-related symptoms or subthreshold mood concerns, particularly when embedded in fiber-rich, Mediterranean-style dietary patterns. Effects should be framed as modest and mechanism-guided, attending to strain/substrate specificity, duration (≥4–8 weeks in many trials), and baseline diet/microbiome [12,17,18,19,20,51,52,53,54,55,61,62,63,64,65]. Dietary strategies and fermented foods may complement such approaches; FMT remains investigational for neurobehavioral indications and requires further safety/efficacy clarification [194,195,196,197,211,212,213].

Future directions (precision and life-course perspectives): Priority areas include (1) longitudinal cohorts beginning in the perinatal period to map sensitive windows and durability of effects; (2) head-to-head RCTs comparing named strains/substrates with adequate dosing and standardized endpoints; (3) biomarker-anchored trials integrating multi-omics, neuroimaging, and endocrine/immune panels; and (4) explicit modifier analyses (dietary fiber, circadian/sleep health, psychotropics, and baseline microbial function) to identify responders [206,207,208,209,210,214,215,216,217,218,219,220,221,222,223,224,225,226,227,228,229,230,231,232]. Additional methodological and translational perspectives underscore these priorities and highlight opportunities for standardized, mechanism-anchored trials [233,234,235]. Advancing precision therapeutics will require integrating host genetics, immune signatures, and microbial biomarkers to tailor interventions for insomnia/circadian disturbance, mood disorders, neurodevelopmental syndromes, and neurodegenerative diseases [64,66,189,208,209,210,214,215,218,219,220,224,225,226,227,228,229,230,231,232]. Named targets such as *Lactobacillus reuteri* and butyrate-linked pathways are attractive candidates for mechanism-first clinical trials [236].

## 5. Limitations

This review has limitations at both the evidence and methodological levels. At the evidence level, many included studies feature modest sample sizes and short intervention periods, with heterogeneous endpoints (different scales and composite measures) that reduce cross-study comparability and preclude reliable meta-analytic aggregation. Strain/substrate specification and dosing are inconsistently reported, and potential effect modifiers—including baseline diet and fiber intake, baseline microbiome features, sleep/circadian factors, medication use, and physical activity—are variably measured or controlled, complicating attribution of effects and generalizability across populations [93,94,95,96,97,98,99,100,101,102,103,104,105,106]. For prebiotics, dose–response and substrate specificity remain insufficiently standardized; for probiotics, strain-specific effects caution against genus- or species-level generalizations [51,52,53,54,55,61,62,63,64,65,78,79,80,81,82,83,84,85,86].

At the methodological level, this is a narrative (non-systematic) review without meta-analysis; we used a PRISMA-style flow for transparency rather than a preregistered systematic protocol. Screening proceeded in two stages (titles/abstracts → full-text), with reasons for exclusion recorded; however, the narrative design may still introduce selection and publication bias, and our English-language focus and database coverage may have missed relevant studies. We excluded in vitro/cell-line-only evidence from study selection/data extraction, considering such work only as background mechanistic context; while appropriate for organism-level outcomes, this choice may underrepresent early molecular insights and thus should be borne in mind when interpreting mechanistic breadth. Finally, variability in microbiome analytical pipelines across studies (sampling, sequencing, and bioinformatic workflows) introduces additional method heterogeneity, which we reflect in our cautious, mechanism-anchored synthesis and in the priorities we outline for future trials [93,94,95,96,97,98,99,100,101,102,103,104,105,106]. These constraints collectively argue for adequately powered, longer-duration, strain/substrate-precise trials, harmonized core outcome sets, and biomarker-anchored designs that enable responder analyses and stronger causal inference in human populations [12,17,18,19,20,51,52,53,54,55,61,62,63,64,65,78,79,80,81,82,83,84,85,86,93,94,95,96,97,98,99,100,101,102,103,104,105,106].

## 6. Conclusions

The gut microbiome plays a foundational and multi-layered role in shaping mood, cognitive function, and decision-making, operating through an intricate interplay of neurochemical, immune, and endocrine pathways. Across diverse lines of evidence, from molecular studies to clinical trials, the microbiome–gut–brain axis has emerged as a compelling target for therapeutic innovation in neuropsychiatry and behavioral health.

Although microbiome-targeted interventions, such as probiotics, psychobiotics, diet modification, and fecal microbiota transplantation, show considerable promise, the field still faces significant challenges. These include the need for rigorous experimental designs, harmonized analytical methodologies, and the development of translational frameworks that can reliably convert correlative findings into mechanism-based, clinically actionable therapies. In practical terms, the leading probiotics and prebiotics highlighted here, together with their putative mechanisms, offer near-term candidates for standardized, mechanism-anchored clinical testing.

A systems-level approach that integrates personalized medicine, multi-omics technologies, and ethical oversight will be essential for advancing the microbiome’s potential in the prevention and treatment of affective, cognitive, and decision-related disorders.

## Figures and Tables

**Table 1:** Probiotics and prebiotics cited in the review: exemplar strain/substrate, putative mechanisms, and most consistent human outcomes.

Category	Exemplar (Strain/Substrate)	Primary Mechanisms	Human Outcome Domains	References
Probiotic	*Lactobacillus rhamnosus* GG (ATCC 53103)	Barrier reinforcement (tight junctions); HPA-axis attenuation; immunomodulation; GABAergic signaling (preclinical)	Stress/anxiety indicators; mood; gastrointestinal symptoms	[17,18,19,20,21]
Probiotic	*Lactobacillus plantarum* PS128	Neurotransmitter modulation (dopamine/serotonin, preclinical); anti-inflammatory actions; SCFA shifts	Mood/stress; exercise performance and recovery	[17,18,19,20,21]
Probiotic	*Bifidobacterium longum* 1714	Vagal signaling; cortisol modulation; attentional/cognitive effects (human experimental evidence)	Perceived stress; attention/memory; mood	[17,18,19,20,21]
Probiotic	*Saccharomyces boulardii*	Barrier/trophic effects; pathogen exclusion; anti-inflammatory pathways	GI symptom improvement; adjunct benefits potentially impacting mood via gut symptom relief	[17,18,19,20,21]
Prebiotic	Inulin-type fructans	Bifidogenic effects; fermentation to SCFAs (butyrate); immune modulation	Mood/stress-related measures; GI function; metabolic/inflammatory markers	[52,53,54,55,56,57,58,59,61,62]
Prebiotic	Galacto-oligosaccharides (GOS)	Bifidogenic; SCFA production; HPA-axis/cortisol modulation (human experimental evidence)	Anxiety- and stress-related measures; attentional bias	[52,53,54,55,56,57,58,59,61,62]
Prebiotic	Resistant starch (types 2–3)	Marked butyrate production; improved barrier integrity; bile-acid signaling	Stress resilience proxies; metabolic and inflammatory markers; GI function	[52,53,54,55,56,57,58,59,61,62]
Prebiotic	Arabinoxylans	Fermentation to SCFAs; immunomodulation; bile-acid interactions	Immune/metabolic markers; select mood/stress proxies	[52,53,54,55,56,57,58,59,61,62]
Prebiotic	β-Glucans	Innate immune modulation (e.g., Dectin-1); SCFA-related effects via co-fermentation	Stress/fatigue; immune function; inflammatory markers	[52,53,54,55,56,57,58,59,61,62]

## Data Availability

No new data were created or analyzed in this study. Data sharing is not applicable to this article.
